# Supplementary material for: Clinicopathologic Analysis of Sinonasal Inverted Papilloma, with Focus on Human Papillomavirus Infection Status
Source: Diagnostics (Basel). 2022 Feb 10;12(2):454. doi: 10.3390/diagnostics12020454 (PMC8871026; doi:10.3390/diagnostics12020454)
Supplement: Supplementary file 1 [file diagnostics-12-00454-s001.zip › Supplemental_Figure.pdf]

Supplemental Figure.

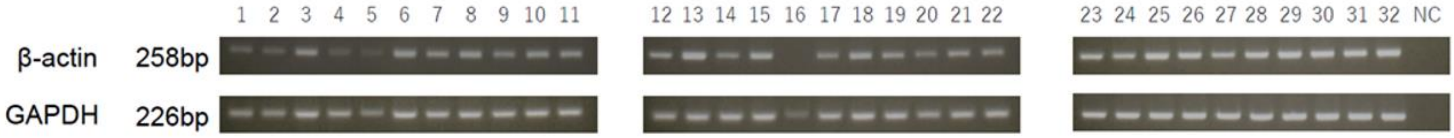

Supplemental Figure S1. The internal control gene amplification. β-actin (258 bp) was amplified in 31 of 32 cases, and GAPDH (226 bp) was amplified in all cases. NC, negative control.
